# Development and Psychometric Evaluation of the Psychological First Aid Competence (PFAC) Scale for Emergency Responders: A Cross‐Sectional, Sequential‐Exploratory Mixed‐Methods Study

**DOI:** 10.1002/hsr2.72202

**Published:** 2026-05-10

**Authors:** Nahid Rajai, Milad Ahmadi Marzaleh, Abbas Ostadtaghizadeh, Maryam Azizi

**Affiliations:** ^1^ Critical Care Nursing department, Faculty of Nursing Aja University of Medical Sciences Tehran Iran; ^2^ Department of Health in Disasters and Emergencies, Health Human Resource Research Center, School of Health Management and Information Sciences Shiraz University of Medical Sciences Shiraz Iran; ^3^ Health in Disasters and Emergencies Department, School of Public Health, Climate Change and Health Research Center (CCHRC) Tehran University of Medical Sciences Tehran Iran; ^4^ Health in Disasters and Emergencies Department, Faculty of Nursing Aja University of Medical Sciences Tehran Iran

**Keywords:** competence, crises, disasters, emergency responders, mental health, psychological first aid

## Abstract

**Background and Aims:**

There is no psychometrically sound tool tailored to assess emergency responders' competence in delivering Psychological First Aid (PFA). This study aimed to develop and evaluate the Psychological First Aid Competence (PFAC) scale for emergency responders.

**Methods:**

We used a sequential‐exploratory mixed‐methods design with a cross‐sectional quantitative phase conducted in Iran (2024). A literature‐derived coding matrix (WHO/IASC/RAPID‐PFA) guided qualitative content analysis of 24 semi‐structured interviews with emergency responders from prehospital services, hospital emergency departments, forensic centers, and disaster response units. Content validity was assessed by 15 experts using item impact score (IIS), content validity ratio (CVR), and item‐level content validity index (I‐CVI). The quantitative phase surveyed 238 responders across diverse emergency settings. Factorability was supported (KMO = 0.799; Bartlett's χ²(351) = 1566.08, *p* < 0.001). Exploratory factor analysis used maximum‐likelihood extraction with Promax rotation. Internal consistency was examined using Cronbach's α with 95% confidence intervals. Items were rated using a 5‐point Likert scale (0–4).

**Results:**

The initial pool (93 items) was refined to 88 and, after item reduction, to a final 27‐item scale across three factors: ethical and belief‐based responses (10 items), psychological responses (10 items), and psychological reactions of victims (7 items), explaining 36.94% of variance. Content validity met a priori threshold (all CVR ≥ critical value; I‐CVI ≥ 0.78). Reliability was good for the total scale (α = 0.85, 95% CI: 0.84–0.86) and acceptable for subscales (α = 0.83; 0.78; 0.81). Nine items were interview‐derived, and 18 were literature‐derived.

**Conclusion:**

The PFAC demonstrates acceptable validity and reliability for assessing PFA competence in emergency responders. Further studies should examine test–retest reliability and confirmatory factor analysis in diverse settings.

## 1 Introduction

Traumatic events, such as natural disasters, severe illnesses, and violence, affect about 70% of the population [1]. According to the American Psychiatric Association, a traumatic event is defined as an experience, witnessed or encountered by an individual, that involves actual or threatened death, serious injury, or a threat to the physical integrity of oneself or others [2]. While some individuals adapt over time, others may face lasting problems like depression, anxiety, substance abuse [3], and post‐traumatic stress disorder (PTSD) [4]. Due to the severe consequences of PTSD such as decreased productivity, diminished work quality, impaired psychological well‐being, disability, and early mortality [5, 6, 7], implementing early and supportive interventions to reduce acute distress and prevent progression to chronic conditions is widely recommended [8]. Given that emergency responders routinely encounter traumatic scenes, their psychological exposure may be both direct and cumulative, making early supportive interventions particularly relevant for this group. Recent disasters in Iran have further highlighted that the psychological impact on emergency response teams—who themselves are exposed to trauma—has often been overlooked and requires urgent attention [9].

Psychological first aid (PFA) is a compassionate intervention offered to individuals in distress during or immediately after a disaster, providing essential support and assistance [10, 11]. Contemporary PFA practices are largely guided by international frameworks such as the WHO PFA Guide [12], IASC MHPSS Guidelines [10], and the Johns Hopkins RAPID‐PFA model [13]. The concept was originally developed in response to acute psychological distress observed among soldiers during World War II [14]. Evidence from emerging studies suggests that PFA interventions may offer beneficial and supportive effects for both disaster‐affected individuals and responder [15]. This intervention enables non‐specialized individuals, like public health professionals and first responders, to recognize and address the needs of those affected [16]. PFA plays key roles in disaster situations by identifying immediate needs, providing disaster information, connecting individuals to social support networks, and fostering resilience [17]. To strengthen the conceptual clarity, we clarified that recent reviews—including that of Wang et al.—show that PFA demonstrates promising, yet still emerging evidence, regarding its effectiveness, particularly in reducing acute distress, and improving supportive communication skills [8].

Emergency responders need strong psychological skills and competence due to their exposure to stressful and traumatic situations. However, many struggle with inadequate psychological preparedness [18]. To address the reviewer's concern regarding undefined concepts, we added a definition: psychological preparedness refers to the cognitive, emotional, and behavioral readiness that enables responders to anticipate, interpret, and manage the psychological demands of crisis situations [18]. A study found that 81% of emergency personnel, including paramedics, lacked adequate training to handle emergencies, which is essential [19]. As Johnson (1977) emphasized, systematic gaps in preparedness during crises can compromise the care quality and be regarded as a form of organizational negligence [20]. Lack of preparedness can reduce the quality and effectiveness of PFA [18]. Research on the effects of PFA training for emergency personnel indicates that trained responders experience fewer psychological consequences, exhibit stronger behavioral skills, and have greater confidence in assisting adults and children compared to untrained volunteers and responders [13, 21, 22, 23, 24]. Taken together, these findings highlight the importance of assessing the responders' psychological competencies, including their readiness to provide PFA effectively.

Evaluating the emergency responders' psychological competencies and skills is essential for enhancing their performance and psychological readiness. Competency refers to the integrated set of knowledge, skills, attitudes, and behavioral capacities that enable responders to perform tasks effectively under crisis conditions [25]. In the context of PFA, competency includes the ability to recognize acute stress reactions, provide immediate emotional and practical support, maintain psychological safety, communicate empathetically, and uphold cultural and ethical considerations when assisting the affected individuals [26]. Developing precise scales for measuring competencies in PFA can help identify service providers' strengths and weaknesses, laying the groundwork for empowerment programs. Despite the centrality of these competencies in emergency response, no existing instrument offers a comprehensive and context‐sensitive assessment specifically tailored to PFA performance in real‐world crisis settings. However, current research shows that the existing scales, while addressing some psychological skills, often fail to meet the specific needs of emergencies and crises and do not provide a multidimensional assessment of the required competencies [27].

Several studies have attempted to develop tools related to psychological preparedness and resilience in disaster settings. Psychological preparedness and disaster resilience, although conceptually related to responders' functioning, are distinct from the task‐specific competencies required to deliver psychological first aid (PFA). Mao et al. (2020) conducted a methodological study to review and classify existing instruments for assessing adult disaster resilience. Their analysis identified eight major domains—optimism, altruism, disaster preparedness, social support, directed control, self‐efficacy, coping strategies, and positive meaning—and highlighted the need for instruments tailored to specific responder populations [28]. These tools focus primarily on general adaptive capacities rather than the immediate interactional skills central to PFA practice. In a subsequent study, Mao et al. (2021) performed a psychometric evaluation of a Disaster Resilience Measurement Instrument among 936 healthcare rescuers from the Sichuan Disaster Response Team. Their findings indicated that the instrument had acceptable validity and reliability for assessing responders' resilience; however, the authors emphasized the need for further validation in different cultural and operational contexts [29]. To improve coherence, we clarified that while resilience instruments assess broad psychological strengths, they do not capture competencies related to crisis communication, ethical conduct, safety decision‐making, or recognition of acute stress reactions, as the core domains of PFA.

Although these studies provide valuable insights into responders' psychological functioning, they do not address the multidimensional and situationally specific competencies needed for delivering PFA. Given the absence of a validated PFA‐specific competency instrument and the demonstrated limitations of existing tools, there is a clear need for a scale grounded in both empirical evidence and field‐derived insights. Accordingly, the final paragraph was revised to present a clearer narrative link to the study's purpose: this study therefore aimed to develop and validate the Psychological First Aid Competency (PFAC) scale specifically for emergency responders, integrating qualitative and quantitative evidence to ensure conceptual relevance and psychometric rigor. The PFAC enables accurate assessment of psychological competencies, supports training program development, and enhances the quality of PFA in crisis settings.

## 2 Methods

### 2.1 Design

This study employed a cross‐sectional design and a sequential‐exploratory mixed‐methods approach in Iran in 2024. Item generation utilized a deductive‐inductive method, beginning with a structured literature review on PFA (deductive) and a qualitative study with emergency responders (inductive) to clarify the concept of competence in delivering psychological first aid. In this study, “PFA competence” refers to the set of knowledge, skills, and behavioral capacities required to provide supportive, safe, and culturally appropriate psychological assistance during acute emergencies. The deductive phase ensured that item development was anchored in established international frameworks, whereas the inductive phase allowed context‐specific competencies to emerge from field experiences.

The literature review was conducted in PubMed, PsycINFO, Scopus, Web of Science, and Google Scholar (to December 2023), using Boolean strings such as “psychological first aid” AND (competenc OR skill OR scale OR instrument*) AND (training OR evaluation OR framework).

Inclusion criteria were guidelines/frameworks and empirical studies explicitly addressing PFA competencies, skills, or measurement; commentaries and papers without a competency focus were excluded. Key references included the WHO PFA Guide (2011) [12], IASC MHPSS Guidelines (2007) [10], the Johns Hopkins RAPID‐PFA model [13], and recent PFA competency/self‐efficacy instruments [30, 31].

From these sources, we extracted a coding matrix with six domains: (i) ethical–legal conduct and safeguarding, (ii) safety and role boundaries, (iii) cultural and gender sensitivity, (iv) supportive communication and reassurance, (v) recognition of survivors' psychological/physiological reactions, and (vi) responder self‐care. These domains reflect internationally recognized expectations for PFA practice and provide an initial conceptual structure for item generation.

These domains guided the generation of preliminary items, which were subsequently refined and evaluated through a quantitative phase designed to establish the scale's psychometric robustness in line with COSMIN standards [32].

### 2.2 Qualitative Study and Item Generation

Twenty‐four semi‐structured, face‐to‐face interviews were conducted to obtain an in‐depth understanding of responders' competence in providing PFA. Participants were selected using purposive sampling with maximum variation to capture diverse perspectives on PFAC. The sample included nurses, emergency medicine physicians, emergency medical technicians, forensic medicine experts, and policymakers. In total, 24 responders participated: 8 nurses, 5 emergency medical technicians, 4 emergency medicine physicians, 3 forensic medicine experts, and 4 policymakers/managers. Policymakers were included because, although they do not directly deliver PFA, they are responsible for establishing competency expectations, developing PFA protocols and training curricula, and shaping the organizational and regulatory environment in which responders operate. Their insights provided essential system‐level perspectives on what constitutes competent PFA practice and ensured that the scale captured both frontline requirements and organizational standards.

Participants were recruited from various emergency settings, including street and road emergencies, emergency medical centers, and disaster response units. This diversity in profession, gender, and work environment was intended to enhance the comprehensiveness and transferability of the findings.

The interview guide was developed directly from the literature‐derived domain matrix, with probes addressing each competency area (e.g., ethical/legal obligations, safety and role boundaries, culturally sensitive communication, recognition of survivors' psychological reactions, and responder self‐care). This ensured that all major domains from the international literature were systematically explored while allowing new categories to emerge inductively from the Iranian context.

Interviews lasted 45–60 min. Each interview started with a general question like “What recent experiences have you had with disasters and emergencies?” The follow‐up prompts included “What strategies do you use to manage stress at the scene of an accident?” At the end of each interview, the recordings were promptly transcribed. Data were analyzed using Lundman and Graneheim's (2004) five‐step conventional content analysis, with guidance from the literature‐based coding (including WHO guidelines, national PFA manuals, and prior empirical studies). The analysis proceeded as follows:

Qualitative data were analyzed using Lundman and Graneheim's (2004) conventional content analysis. First, all interview transcripts were read several times to achieve immersion and obtain a comprehensive sense of the data. Meaning units relevant to PFA competence were then identified and subsequently condensed and assigned initial codes. These codes were continuously compared with the predefined categories in the literature‐based coding matrix; when meaning units did not align with existing categories, new categories were developed inductively. Through an iterative process, the codes and categories were reviewed, refined, and organized into overarching themes that directly informed the generation of questionnaire items. Throughout this process, emerging data were systematically compared with international PFA guidelines (WHO, IASC, RAPID‐PFA) to ensure conceptual fidelity while preserving culturally grounded content [33].

To ensure fidelity to international PFA models, the interview guide and coding matrix were anchored in the WHO PFA Guide, IASC MHPSS Guidelines, and the RAPID‐PFA framework. During analysis, all emergent codes were systematically compared with these domains, and only those consistent with recognized PFA principles were retained for item development. Divergent personal practices reported by responders were excluded unless later confirmed as consistent by expert reviewers during the CVR/CVI validation stage. This process ensured that the final items reflected competencies aligned with standard PFA while still incorporating culturally relevant expressions.

To ensure the validity and reliability of the data, factors such as credibility, dependability, transferability, and confirmability were considered [34].

Provenance of items. An initial pool was created based on both literature‐derived domains and interview‐derived codes. The initial item pool consisted of 93 items generated through this combined deductive–inductive process before undergoing face and content validity assessment. For example, nine final items (Items 5, 6, 7, 11, 12, 13, 14, 20, and 26) originated inductively from responders' narratives, while the remaining items were deductively derived from the literature review (e.g., WHO, IASC, RAPID‐PFA, and related psychometric studies).

In designing the items, attention was paid to aspects such as clarity of meaning, respondents' ability to understand and answer the questions, simplicity and fluency, lack of ambiguity, brevity, positive wording, cultural relevance, and comprehensive coverage of all PFAC scale dimensions [35]. The research team then reviewed the item pool in several stages, leading to the removal of duplicate items and the merging of those with similar meanings. Ultimately, the initial PFAC scale consisted of 81 items, using a five‐point Likert scale (never = 0, rarely = 1, sometimes = 2, often = 3, always = 4), and proceeded to the psychometric evaluation phase.

### 2.3 Quantitative Study and Item Reduction

At this stage, to determine the psychometric properties of PFAC according to the COSMIN criteria [36], we used content validity, construct validity, and reliability.

To evaluate the psychometric properties of the PFAC scale, the quantitative phase was conducted between February and May 2024. Data were collected using an anonymous, self‐administered electronic questionnaire distributed through professional emergency response networks, organizational mailing lists, and emergency care communication channels across multiple provinces in Iran. A maximum variation sampling strategy was used to capture responders with diverse demographic and occupational characteristics, including differences in age, gender, education level, professional roles, years of experience, and exposure to various types of emergencies and disasters.

A total of 238 responders completed the questionnaire. Inclusion criteria were: (i) at least 1 year of professional experience in emergency response; (ii) direct involvement in disaster or emergency operations; (iii) the ability to read and respond to the questionnaire independently; and (iv) willingness to participate. Individuals without direct field experience or who were unable to provide informed consent were excluded. Participants represented a broad range of emergency settings, including prehospital emergency services, hospital emergency departments, forensic medicine centers, and disaster response units.

The demographic and occupational characteristics of the quantitative sample—including age, gender, educational level, professional role, work setting, and years of experience—are presented in Table 2. This diversity was intended to enhance the generalizability and applicability of the PFAC scale across different emergency response contexts. This heterogeneity enhances the representativeness of the sample and strengthens the generalizability of the PFAC scale across different emergency response contexts.

#### 2.3.1 Face Validity

Face validity refers to the degree of alignment between the scale and the subject being measured, as perceived by respondents [37]. In other words, content validity involves an objective judgment about the scale's structure and answers the question: “Do non‐expert individuals (the target group) perceive it the same way the researcher intends?” [38]. To assess content validity, we employed both qualitative and quantitative methods. In the qualitative approach, the initial PFAC questionnaire was given to eight crisis responders, who were asked to evaluate the items based on their difficulty level, relevance, and ambiguity. After confirming qualitative content validity and revising unsuitable items, the Item Impact Score quantitative method was employed to eliminate inappropriate items and assess each item's relevance to the overall scale [39]. Each item was rated using a five‐point Likert scale: completely appropriate [5], somewhat appropriate [4], moderately appropriate [40], slightly appropriate [41], and completely inappropriate [40]. The goal was to assess the relevance of each item for measuring PFAC in responders. The item impact score was then calculated using the following formula [38].

ItemImpactScore=Frequency(%)×Suitable

If an item's impact score exceeded 1.5, it was deemed suitable for further analysis and retained [42]. The threshold of 1.5 was based on an average score of 3 and a frequency mean of 50% [35]. After removing unsuitable items, we conducted the next phase, which focused on content validity.

#### 2.3.2 Content Validity

Content validity was examined through both qualitative and quantitative methods. For the qualitative assessment, 15 experts in psychology, psychiatry, disaster and emergency health, civil defense, and crisis management provided feedback on the wording, grammar, item allocation, and scaling. Quantitative content validity was then determined using two indices based on expert feedback: The content validity ratio (CVR) and the content validity index (CVI). The CVR assessed the essentiality of each item, while the CVI verified the relevance of each item in accurately measuring PFAC among responders [38].

The content validity ratio (CVR) was assessed by distributing a questionnaire among 15 experts, who rated each item as “essential,” “useful but not essential,” or “not essential.” The CVR for each item was then computed, using the following formula:

$$CVR=\frac{nE-\frac{N}{2}}{\frac{N}{2}}.$$

In this formula, *nE* represents the number of evaluators who considered the item essential, and *N* represents the total number of evaluators who reviewed the item. The resulting value was then compared to those in Lawshe's CVR table. If the calculated CVR exceeded the table value, it indicated that the item was essential and significant [35].

To assess the CVI, evaluators were asked to examine each item for its relevance to PFAC in emergency responders, rating their opinions on a four‐point Likert scale (1: Not relevant, 2: Somewhat relevant, 3: Relevant but needs revision, 4: Highly relevant). The content validity index (CVI) was then calculated using the following formula [38].

$$\text{CVI}\;=\;\frac{\text{The}\;\text{number}\;\text{of}\;\text{evaluators}\;\text{who}\;\text{gave}\;\text{each}\;\text{item}\;a\;\text{score}\;\text{of}\;3\;\text{and}\;4}{\text{Total}\;\text{number}\;\text{of}\;\text{evaluators}}.$$

A score of 0.78 or higher was set as the cutoff for the CVI, indicating acceptance and retention of the items. A score between 0.70 and 0.78 required item revision, while a score below 0.70 indicated item removal.

#### 2.3.3 Item Analysis

Item analysis is a preliminary evaluation of the scale within the target population, conducted before assessing construct validity. In this method, the correlation of each item with others (inter‐item correlation) is examined. Each item is expected to show a correlation of at least 0.30 with at least two other items; otherwise, it is removed. Additionally, if two items have a correlation above 0.70, they are compared for semantic similarity; if they measure the same concept, one is eliminated, whereas dissimilar items with high correlations are retained because such correlations may occur by chance [43].

#### 2.3.4 Construct Validity

Construct validity reflects how effectively a scale measures its intended concept and is commonly applied in self‐report and subjective scales. Various methods can assess construct validity, with this study using Exploratory Factor Analysis (EFA). Factor analysis is a multivariate approach designed to examine correlations among observed variables and to identify the underlying factors, latent variable, or internal attributes. Because scales may contain redundant items that reduce analytic efficiency, only items that truly differentiate members of the population should be evaluated [44]. Accordingly, EFA was used to clarify the relationships among the items and to simplify the scale by identifying coherent clusters of interrelated items.

To perform the EFA, we entered the data from electronically completed questionnaires into SPSS. Score distributions were first examined using skewness (±3) and kurtosis (±7) criteria. Sample size adequacy was then assessed. Although a minimum sample of 200 is commonly recommended for EFA, we also considered psychometric power recommendations from prior literature [45, 46], which indicates that samples above 200 provide ≥ 0.80 power to detect factor loadings of 0.40 or greater in multifactor models. Based on these recommendations, our sample of 238 responders was deemed sufficient to ensure both statistical power and stable factor solutions.

Sampling adequacy was further evaluated using the Kaiser–Meyer–Olkin (KMO) index, which yielded a value of 0.799, indicating acceptable adequacy. Bartlett's test of sphericity confirmed that correlations among items were sufficient for factor extraction. Outliers and missing data were examined, and missing values were replaced using the median.

Given the approximate normal distribution of the data, maximum likelihood extraction was used to identify latent factors. Eigenvalues ≥ 1 were used as the initial criterion for factor retention, and factors below this threshold were excluded. Because initial unrotated solutions are often difficult to interpret, both Varimax (orthogonal) and Promax (oblique) rotations were applied sequentially to obtain interpretable factor structures.

#### 2.3.5 Reliability

Reliability in this study was assessed through the internal consistency method, which examines the extent to which items within a scale measure the same underlying construct. To evaluate this internal agreement, Cronbach's alpha coefficients were calculated for the overall scale and for each extracted factor. According to psychometric standards, an alpha value of 0.70 or higher is generally considered acceptable [38]. Therefore, items that reduced the internal consistency of their respective subscales were reviewed, and none met criteria for removal, as all retained items contributed meaningfully to the reliability structure.

### 2.4 Data Analysis

All quantitative analyzes were performed using IBM SPSS Statistics version 26 (IBM Corp., Armonk, NY, USA). Descriptive statistics were reported as means and standard deviations (SDs) for normally distributed variables, or medians and interquartile ranges (IQRs) for non‐normally distributed variables. Exploratory factor analysis was conducted using maximum likelihood extraction with Varimax and Promax rotations, and item‐retention decisions were based on primary loadings ≥ 0.40 and cross‐loadings < 0.30. Reliability analyzes (Cronbach's α with 95% confidence intervals) were also performed in SPSS. Content validity indices (Item Impact Score, CVR, and CVI) were calculated manually in Microsoft Excel version 2019.

Statistical reporting: We prioritized estimation over null‐hypothesis significance testing. Continuous variables are summarized as means ± SD (or medians [IQR] when non‐normal), and categorical variables as n/N (%). Reliability coefficients (Cronbach's α) are presented with 95% confidence intervals. When *p* values are reported, they are accompanied by the corresponding test statistics and degrees of freedom and formatted according to journal guidelines (e.g., *p* < 0.001; values between 0.001 and 0.01 to the nearest thousandth; ≥ 0.01 to the nearest hundredth; *p* > 0.99).

#### 2.4.1 Study Design and Statistical References

The sequential‐exploratory mixed‐methods design and the psychometric procedures applied in this study were grounded in recognized methodological references. Guidance from COSMIN for evaluating health measurement instruments [36, 47]; DeVellis for scale development [48]; Kline and Tabachnick & Fidell for multivariate methods [49]; Standards for sampling adequacy and factorability were guided by Kaiser's criteria for KMO [50]; and Bartlett's test of sphericity. Procedures for quantitative content validation drew upon Lawshe and Lynn's recommendations for CVR and CVI [51]; confidence intervals for reliability estimates were calculated following Bonett's approach to Cronbach's α [52]. Full citations are provided in the reference list.

#### 2.4.2 Prespecified Versus Exploratory Analyzes

The analyzes planned a priori and included the assessment of face and content validity (IIS, CVR, CVI), item analysis, and exploratory factor analysis using maximum likelihood extraction, with Promax rotation specified as the final rotation method. Exploratory procedures were restricted to screening dimensionality using PCA and comparing Varimax and Promax rotations to enhance interpretability. No subgroup analyzes were prespecified or performed.

#### 2.4.3 Statistical Tests and Significance (Per SAMPL)

Assessment of statistical assumptions followed standard SAMPL guidance. Normality of the item distributions was examined using skewness and kurtosis criteria (| skew | ≤ 3; |kurtosis | ≤ 7). Sampling adequacy and factorability were evaluated using the KMO index and Bartlett's test of sphericity (χ², df, two‐sided P). Exploratory factor analysis was performed using maximum likelihood extraction, with item‐retention criteria defined as a primary loading of ≥ 0.40, cross‐loading < 0.30, and review of redundancy through inter‐item correlations (flagged at r > 0.70). Content validity was assessed using the Item Impact Score (threshold ≥ 1.5), CVR (Lawshe; critical value for 15 experts), and I‐CVI (cutoffs ≥ 0.78; 0.70–0.77 for revision; < 0.70 for removal). Reliability was evaluated through Cronbach's α with 95% confidence intervals (Bonett). All hypothesis tests were two‐sided with a prespecified significance level of α = 0.05. Analyzes were conducted in IBM SPSS Statistics v26, and auxiliary computations for CVR, CVI, and confidence intervals were performed in Microsoft Excel 2019. Reporting of estimates, uncertainty, and *p*‐values adhered to SAMPL recommendations.

### 2.5 Ethical Considerations

This research was approved by the Biomedical Research Ethics Committee with the code of IR.SUMS.REC.1402.368. Informed consent was obtained from all participants, who were assured that their information would remain confidential and that participation was entirely voluntary.

## 3 Result

### 3.1 Item Generation

The initial item pool, derived from both the structured literature review and the qualitative interview codes, encompassed the six key competency domains identified in the literature—including ethical–legal conduct, safety and role boundaries, cultural and gender sensitivity, supportive communication and reassurance, recognition of survivors' psychological and physiological reactions, and responder self‐care—along with additional categories that emerged inductively from interview data. Altogether, this process yielded 93 preliminary items. Following several rounds of refinement by the research team, during which duplicate or semantically similar items were removed or merged, the pool was reduced to 88 items. After psychometric item reduction, 27 items remained; among these, 9 items (Items 5, 6, 7, 11, 12, 13, 14, 20, and 26) originated directly from responders' narratives, while 18 items were developed deductively based on international guidelines (WHO, IASC, RAPID‐PFA) and previous psychometric/educational studies. This ensured that the PFAC scale was simultaneously grounded in global evidence and sensitive to local context.

The qualitative findings played a critical role in shaping item development. For example, ethical and cultural sensitivity emerged strongly in participants' accounts. One responder noted: *“Female survivors must be counseled by a female psychologist; in one incident, this was ignored and it caused conflict with the survivor's husband.”* This insight informed items addressing gender‐concordant support and culturally respectful practice. Safety considerations also featured prominently. A participant recalled: *“During an earthquake, a responder entered a survivor's home just before an aftershock destroyed the building and injured the responder,”* illustrating the importance of assessing the hazard and informing safety‐related items. Legal considerations were similarly emphasized; A responder stated: *“In emergencies like suicide risk, we must write a detailed report and submit it according to the protocol.”* This narrative guided the development of items related to legal responsibilities and maintaining professional boundaries.

Participants also described concerns related to the appropriateness of interventions. One survivor emphasized the need for non‐intrusive support, stating: *“I just wanted to be alone and cry, but too many teams kept approaching me.”* These informed items aiming to capture sensitivity to survivors' individual coping needs. Emotional and psychological reactions were another major theme. Responders described intense reactions of their own—for example, *“At the crash site of the Ukrainian plane, one of our responders started screaming when confronted with the scene.”* Such accounts contributed to items reflecting recognition of diverse psychological responses in disaster settings.

Together, these qualitative insights were systematically coded and transformed into item statements, ensuring that the PFAC scale captured both theoretically grounded competencies and the realities of frontline practice.

### 3.2 Face and Content Validity

Quantitative and qualitative assessments of face and content validity supported the overall clarity and relevance of the items. *Item Impact Score (IIS)*, *Content Validity Ratio (CVR)*, and *Content Validity Index (I‐CVI)* values are presented in Table 1. Out of the 88 evaluated items, 7failed to meet IIS, CVR, or I‐CVI thresholds and were removed. Also, 81 items were retained for item analysis and factorability assessment. The retained items demonstrated strong essentiality (CVR ≥ critical cutoff) and high relevance (I‐CVI ≥ 0.78), confirming adequate content validity.

**Table 1:** Quantitative evaluation of the questionnaire's face and content validity and item analysis.

No.	Statement	IIS score	CVR	CVI	Correlation with other statements
1	Emotional agitation among disaster survivors during the initial shock phase is to be expected.	3	0.8	0.8	0.51
2	Denial and selective forgetting can temporarily soothe disaster victims, promoting short‐term mental relief.	2.5	0.8	1	0.39
3	People of varying ages exhibit distinct psychological responses to disasters.	2.4	0.9	1	0.45
4	Within hours after an incident, individuals may experience heightened feelings of forgiveness and sacrifice.	2.3	1	1	0.48
5	Effective coordination of assistance provided by disaster survivors is essential.	1.8	1	0.9	0.38
6	Temporary periods of calm and happiness emerge for survivors within a week to a few months post‐disaster.	1.95	1	0.9	0.5
7	Two to three months after an event, people gradually become aware of the full scope of the damages.	3.3	1	0.8	0.43
8	Six months to a year later, survivors initiate psychological healing and reconstruct their lives.	2.8	1	0.8	0.39
9	During the reality‐check phase, survivors often need intensified psychological support.	2.5	1	0.8	0.42
10	Disaster survivors exhibit a variety of physiological reactions.	3.8	1	0.8	0.44
11	In the shock phase, disaster victims require special attention due to diminished reasoning and functional capacities.	3.5	0.8	0.8	0.52
12	I encourage isolated individuals to engage in conversation and join group settings.	2.5	0.8	0.8	0.61
13	Distraction techniques are effective in calming frightened individuals during crises.	3.5	0.7	1	0.44
14	Disaster victims display a range of psychological responses.	3	0.7	1	0.54
15	PFA involves identifying the needs and concerns of disaster victims.	2	0.8	1	0.35
16	A majority of disaster survivors experience psychological disturbances.	3.2	0.8	1	0.32
17	Most affected individuals require mental health services.	2.2	0.8	1	0.48
18	Many disaster survivors recover from distress through self‐support and autonomy.	3.8	1	1	0.38
19	I administer PFAimmediately upon encountering highly distressed individuals.	3.34	1	1	0.44
20	I offer PFA in any sufficiently safe environment.	3	1	1	0.42
21	The goal of PFAis to support newly affected individuals.	2.5	1	1	0.43
22	PFAis an essential scale for survivors' long‐term psychological rehabilitation.	3	1	1	0.52
23	PFAprovides a valuable alternative to debriefing.	2.8	1	1	0.42
24	To promote a sense of calm in victims, I avoid pressuring them to discuss their emotions.	2.5	1	0.9	0.33
25	I prioritize the comfort and well‐being of disaster survivors.	2.5	1	0.8	0.43
26	I assist individuals who need information and social support.	2.8	1	0.8	0.52
27	PFAis intended for disaster survivors in distress.	2.3	1	1	0.44
28	It also serves individuals in urgent need of advanced support.	2.6	0.8	1	0.42
29	In a crisis, I provide aid to all disaster survivors, regardless of their willingness.	2.8	0.8	1	0.35
30	PFA interventions should be carried out exclusively by trained psychologists.	2.4	0.7	1	0.35
31	I believe survivors do not need to be informed of the disaster's full details.	2.4	0.8	1	0.46
32	To foster calm and security, I make realistic promises only.	2.6	1	1	0.4
33	I obtain permission before administering any form of care.	3.5	1	1	0.42
34	I maintain a calm demeanor while providing psychological first aid.	3.5	1	1	0.54
35	I demonstrate understanding of the survivors' circumstances.	4	1	1	0.48
36	I believe there is no need to explain traumatic details to adolescents.	2.3	0.8	1	0.42
37	I refrain from exaggerating my skills to instill confidence in survivors.	2.5	1	1	0.38
38	I share other survivors' stories to alleviate feelings of isolation among victims.	2.4	1	1	0.36
39	I offer constructive advice on survivors' conduct during a disaster.	3.4	1	1	0.42
40	I thoroughly assess available resources and services.	3.6	1	1	0.44
41	I help victims frame their experience (e.g., “You're fortunate; it could've been worse”).	3.8	1	0.8	0.46
42	I inquire about survivors' concerns, even if they seem obvious.	2.5	0.8	0.8	0.44
43	I provide information only when certain of its accuracy.	2.8	1	0.9	0.46
44	I offer reassurances to survivors as appropriate (e.g., “Your home will be rebuilt soon”).	3	0.8	1	0.42
45	For individuals reluctant to discuss their experiences, I offer supportive gestures (like providing water).	3.8	0.8	1	0.52
46	I provide space for survivors to share their experiences only if they wish to do so	2.8	0.8	1	0.34
47	I attend closely to body language when interacting with survivors.	3.5	0.8	1	0.42
48	I involve survivors in tasks they are capable of performing.	2.4	1	1	0.36
49	I refrain from criticizing survivors for their mistakes.	3.4	1	1	0.42
50	I highlight survivors' good fortune to instill a sense of calm.	3.6	1	1	0.44
51	I promptly inform survivors of any changes in available services or conditions.	3.5	1	1	0.54
52	I ensure that children remain under the care of supportive adults.	4	1	1	0.48
53	I assist in reuniting survivors with their families.	2.3	0.8	1	0.42
54	I can engage in supportive listening with cultural sensitivity.	2.5	1	1	0.38
55	I consider empathetic listening and understanding as highly effective for calming survivors.	2.4	1	1	0.36
56	I recognize silence as a potentially effective intervention.	3.4	1	1	0.42
57	I prioritize attention for individuals exhibiting self‐harming or harmful thoughts.	3.6	1	1	0.44
58	I identify and assist those needing special attention.	2.5	0.8	0.8	0.44
59	I am aware of potentially harmful language to avoid with survivors.	3.4	1	1	0.46
60	I avoid actions that could cause further harm to survivors.	3.5	1	1	0.43
61	I emphasize preserving the dignity of sexual violence survivors.	3.6	1	1	0.45
62	I take measures to prevent further harm to survivors.	3.3٭	1	0.9	0.42
63	I view assisting survivors as a legal and ethical obligation, regardless of their willingness.	3.8	1	1	0.51
64	I am attentive to the legal and cultural contexts of survivors in crises.	4	1	1	0.49
65	I take precautions to prevent individuals from self‐harm.	3.7	1	1	0.44
66	I assess the safety of the scene before intervening, even in emergencies.	3.6	1	1	0.47
67	I ensure nondiscriminatory and fair access to services.	3.9	1	1	0.52
68	I take precautions to prevent physical injuries to survivors.	3.4	1	0.9	0.43
69	I take precautions to prevent psychological harm to survivors.	3.6	1	1	0.48
70	I remain unbiased toward survivors' beliefs.	3.5	1	1	0.45
71	I adopt culturally respectful attire to gain trust and respect.	4	1	1	0.50
72	I communicate in the language of the survivors.	3.7	1	1	0.49
73	Female survivors receive support from female facilitators.	3.5	1	1	0.46
74	I respect the religious beliefs of survivors.	3.8	1	1	0.52
75	I consider cultural norms when holding a survivor's hand.	3.4	1	1	0.44
76	I consider cultural norms when placing a hand on a survivor's shoulder.	3.6	1	1	0.47
77	I align my actions with the survivor's beliefs.	3.3	1	1	0.43
78	I prioritize self‐care while assisting during crises.	3.7	1	1	0.45
79	I balance attention between myself and the survivor.	3.8	1	1	0.48
80	I delay personal needs until the crisis is resolved.	3.6	1	1	0.49
81	I believe discussing crisis conditions with colleagues is necessary.	2.4	1	1	0.36
82	I am aware of other on‐site emergency response activities.	3.4	1	1	0.42
83	I avoid interfering with search and rescue personnel	0.6٭			
84	I am aware of my role boundaries.	0.9٭			
85	I stay informed of emergency responses and support resources.	0.9٭			
86	I follow instructions from crisis management authorities.	0.9٭			
87	I am prepared to support disaster survivors.	0.9٭			
88	I identify and assist individuals from religious and ethnic minorities.	3.4			

*Note:* IIS = Item Impact Score (threshold ≥ 1.5); CVR = Content Validity Ratio (Lawshe, critical value for *N* = 15 experts); I‐CVI = Item‐level Content Validity Index (cutoffs: ≥ 0.78 accept; 0.70–0.77 revise; < 0.70 remove). Correlation refers to inter‐item (redundancy flagged at r > 0.70). Asterisks (*) denote items removed in subsequent steps.

### 3.3 Item Analysis

Inter‐item correlations confirmed adequate homogeneity while also identifying redundant relationships. No pair of items exceeded the r > 0.70 redundancy threshold. All retained items correlated at least 0.30 with at least two other items, supporting internal coherence within the item pool.

### 3.4 Construct Validity

Exploratory Factor Analysis (EFA) was performed on data from 238 responders (demographic characteristics shown in Table 2).

**Table 2:** Summary of demographic characteristics of responders in the quantitative phase (*n* = 238).

Demographic variable	Category	Frequency (*n*)	Percentage (%)
Gender	Female	78	32.8
	Male	157	66
	Missing data	3	1.3
Education level	Bachelor's degree	108	45.4
	Master's degree	42	17.6
	Doctoral degree	9	3.8
	Associate degree	69	29
	Missing data	10	4.2
Age (Years)	Mean ± SD	36 ± 7	
Work experience (Years)	Mean ± SD	12 ± 6	

*Note:* Values shown as *n/N* (%). Missing Data are reported as *n/N* (%). Age and work experience reported as mean ± SD.

Sampling adequacy was confirmed by a KMO value of 0.799 and a significant Bartlett's test of sphericity (χ²(351) = 1566.08, *p* < 0.001), indicating that the correlation matrix was suitable for factor analysis (Table 3).

**Table 3:** KMO (Kaiser‐Meyer‐Olkin) measure of sampling adequacy and Bartlett's test results.

KMO and Bartlett's Test		
Kaiser‐Meyer‐Olkin measure of sampling adequacy.		0.799
Bartlett's test of sphericity	Approx. Chi‐Square	1566.075
	Df	351
	Sig.	*p* < 0.001

*Note:* KMO = Kaiser–Meyer–Olkin sampling adequacy (0.799 indicates middling‐to‐good, just below 0.80 “very good”). Bartlett's test of sphericity: χ²(351) = 1566.08, *p* < 0.001.

An initial inspection of eigenvalues identified 20 components with values greater than one; however, many of these components lacked conceptual coherence upon further examination. Through successive rounds of EFA using maximum likelihood extraction followed by Promax rotation, items with low primary factor loadings (< 0.40), substantial cross‐loadings (≥ 0.30), or no meaningful loading on any factor were systematically removed.

This iterative refinement process resulted in a stable and theoretically consistent three‐factor solution consisting of 27 items, which together explained 36.94% of the total variance (Table 4).

**Table 4:** The total variance explained by the factors of the PFAC scale in the initial and final rotations.

Total variance explained							
Component	Initial eigenvalues			Extraction sums of squared loadings			Rotation sums of squared loadings^a^
	Total	% of Variance	Cumulative %	Total	% of Variance	Cumulative %	Total
1	5.385	19.945	19.945	5.385	19.945	19.945	4.246
2	2.491	9.227	29.172	2.491	9.227	29.172	3.888
3	2.100	7.776	36.948	2.100	7.776	36.948	3.590
4	1.354	5.016	41.964				
5	1.237	4.581	46.545				
6	1.062	3.933	50.478				
7	1.052	3.895	54.373				
8	0.992	3.675	58.048				
9	0.973	3.605	61.652				
10	0.852	3.155	64.808				
11	0.828	3.067	67.874				
12	0.814	3.014	70.889				
13	0.759	2.811	73.700				
14	0.720	2.667	76.367				
15	0.704	2.607	78.974				
16	0.662	2.452	81.426				
17	0.619	2.294	83.720				
18	0.590	2.187	85.907				
19	0.582	2.155	88.063				
20	0.531	1.967	90.030				
21	0.488	1.807	91.837				
22	0.434	1.607	93.444				
23	0.422	1.563	95.007				
24	0.387	1.432	96.439				
25	0.337	1.250	97.689				
26	0.322	1.193	98.881				
27	0.302	1.119	100.000				
Extraction Method: Principal Component Analysis.							

*Note:* Initial eigenvalues are from dimensionality screening; the final latent structure was estimated via EFA (maximum likelihood) with Promax rotation. Components with eigenvalues > 1 and interpretability were retained; cumulative variance explained by the final 3‐factor solution = 36.94%.

^a^ When components are correlated, sums of squared loadings cannot be added to obtain a total variance.

The final factors were labeled as Ethical and belief‐based responses (10 items), Psychological responses (10 items), and Psychological reactions of victims (7 items). All retained items demonstrated satisfactory loading magnitudes and conceptual clarity, as shown in Tables 5 and 6.

**Table 5:** The final factors derived from the factor analysis, conducted using the most recent promax rotation, along with the factor loadings for each item.

Pattern matrix	Component 1	Component 2	Component 3
I align my actions with the survivor's beliefs.	0.700		
I emphasize preserving the dignity of sexual violence survivors.	0.683		
I respect the religious beliefs of survivors.	0.674		
I am attentive to the legal and cultural contexts of survivors in crises.	0.664		
I communicate in the language of the survivors.	0.583		
Female survivors receive support from female facilitators.	0.558		
I identify and assist individuals from religious and ethnic minorities.	0.534		
I remain unbiased toward survivors' beliefs.	0.531		
I obtain permission before administering any form of care.	0.454		
I ensure nondiscriminatory and fair access to services.	0.408		
I highlight survivors' good fortune to instill a sense of calm.		0.699	
I help victims frame their experience (e.g., “You're fortunate; it could've been worse”).		0.691	
I share other survivors' stories to alleviate feelings of isolation among victims.		0.660	
I provide space for survivors to share their experiences only if they wish to do so		0.574	
I offer reassurances to survivors as appropriate (e.g., “Your home will be rebuilt soon”).		0.572	
I offer constructive advice on survivors' conduct during a disaster.		0.550	
I inquire about survivors' concerns, even if they seem obvious.		0.506	
In a crisis, I provide aid to all disaster survivors, regardless of their willingness		0.445	
I offer PFA in any sufficiently safe environment.		0.378	
I believe there is no need to explain traumatic details to adolescents.		0.376	
During the reality‐check phase, survivors often need intensified psychological support.			0.758
In the shock phase, disaster victims require special attention due to diminished reasoning and functional capacities.			0.657
Disaster survivors exhibit a variety of physiological reactions			0.644
Disaster victims display a range of psychological responses.			0.638
People of varying ages exhibit distinct psychological responses to disasters.			0.522
Six months to a year later, survivors initiate psychological healing and reconstruct their lives.			0.479
Emotional agitation among disaster survivors during the initial shock phase is to be expected.			0.404

*Note:* Loadings from EFA (maximum likelihood) with Promax rotation; loadings < 0.30 suppressed. Item retention required primary loading ≥ 0.40 and cross‐loading < 0.30. Factors: F1 = Ethical & belief‐based responses; F2 = Psychological responses; F3 = Psychological reactions of victims.

**Table 6:** Labeling of each factor in the scale according to the corresponding items and their respective factor loadings.

Item text	Factor loading	Factor
1. I align my actions with the survivor's beliefs.	0.700	Ethical and belief‐based responses
2. I emphasize preserving the dignity of sexual violence survivors.	0.683	
3. I respect the religious beliefs of survivors.	0.674	
4. I am attentive to the legal and cultural contexts of survivors in crises.	0.664	
5. I communicate in the language of the survivors.	0.583	
6. Female survivors receive support from female facilitators.	0.558	
7. I identify and assist individuals from religious and ethnic minorities.	0.534	
8. I remain unbiased toward survivors' beliefs.	0.531	
9. I obtain permission before administering any form of care.	0.454	
10. I ensure nondiscriminatory and fair access to services.	0.408	
11. I highlight survivors' good fortune to instill a sense of calm.	0.699	Psychological responses
12. I help victims frame their experience (e.g., “You're fortunate; it could've been worse”).	0.691	
13. I share other survivors' stories to alleviate feelings of isolation among victims.	0.660	
14. I provide space for survivors to share their experiences only if they wish to do so	0.574	
15. I offer reassurances to survivors as appropriate (e.g., “Your home will be rebuilt soon”).	0.572	
16. I offer constructive advice on survivors' conduct during a disaster.	0.550	
17. I inquire about survivors' concerns, even if they seem obvious.	0.506	
18. In a crisis, I provide aid to all disaster survivors, regardless of their willingness	0.445	
19. I offer PFA in any sufficiently safe environment.	0.378	
20. I believe there is no need to explain traumatic details to adolescents.	0.376	
21. During the reality‐check phase, survivors often need intensified psychological support.	0.758	Psychological Reactions of Victims
22. In the shock phase, disaster victims require special attention due to diminished reasoning and functional capacities.	0.657	
23. Disaster survivors exhibit a variety of physiological reactions	0.644	
24. Disaster victims display a range of psychological responses.	0.638	
25. People of varying ages exhibit distinct psychological responses to disasters.	0.522	
26. Six months to a year later, survivors initiate psychological healing and reconstruct their lives.	0.479	
27. Emotional agitation among disaster survivors during the initial shock phase is to be expected.	0.404	

The PFAC scale was finalized, consisting of three dimensions and 27 items, as detailed in Table 7. An analysis of the factor loadings indicates that the ethical and belief‐based responses dimension carries the highest factor loading.

**Table 7:** The final PFAC scale.

Dimension	Item number	Statements	Always	Often	Sometimes	Rarely	Never
Ethical and belief‐based responses	1.	I align my actions with the survivor's beliefs.					
	2.	I emphasize preserving the dignity of sexual violence survivors.					
	3.	I respect the religious beliefs of survivors.					
	4.	I am attentive to the legal and cultural contexts of survivors in crises.					
	5.	I communicate in the language of the survivors.					
	6.	Female survivors receive support from female facilitators.					
	7.	I identify and assist individuals from religious and ethnic minorities.					
	8.	I remain unbiased toward survivors' beliefs.					
	9.	I obtain permission before administering any form of care.					
	10.	I ensure nondiscriminatory and fair access to services.					
Psychological responses	11.	I highlight survivors' good fortune to instill a sense of calm.					
	12.	I help victims frame their experience (e.g., “You're fortunate; it could've been worse”).					
	13.	I share other survivors' stories to alleviate feelings of isolation among victims.					
	14.	I provide space for survivors to share their experiences only if they wish to do so					
	15.	I offer reassurances to survivors as appropriate (e.g., “Your home will be rebuilt soon”).					
	16.	I offer constructive advice on survivors' conduct during a disaster.					
	17.	I inquire about survivors' concerns, even if they seem obvious.					
	18.	In a crisis, I provide aid to all disaster survivors, regardless of their willingness					
	19.	I offer PFA in any sufficiently safe environment.					
	20.	I believe there is no need to explain traumatic details to adolescents.					
Psychological reactions of victims	21.	During the reality‐check phase, survivors often need intensified psychological support.					
	22.	In the shock phase, disaster victims require special attention due to diminished reasoning and functional capacities.					
	23.	Disaster survivors exhibit a variety of physiological reactions					
	24.	Disaster victims display a range of psychological responses.					
	25.	People of varying ages exhibit distinct psychological responses to disasters.					
	26.	Six months to a year later, survivors initiate psychological healing and reconstruct their lives.					
	27.	Emotional agitation among disaster survivors during the initial shock phase is to be expected.					

*Note:* Items rated on a 5‐point scale (0 = Never to 4 = Always). Subscale and total scores are sums of constituent items; total score range = 27–135 (higher scores indicate greater PFA competence).

### 3.5 Reliability

Internal consistency reliability was high for the overall scale and acceptable across all three subscales. The total PFAC scale demonstrated a Cronbach's alpha of 0.85 (95% CI: 0.84–0.86), indicating strong internal coherence. The subscales also showed satisfactory reliability, with alpha values of 0.83 for ethical and belief‐based responses, 0.78 for psychological responses, and 0.81 for psychological reactions of victims; the corresponding confidence intervals are detailed in Table 8. Reliability analyzes based on the subsample of 100 responders further confirmed the stability of the scale, as no item deletion improved the alpha coefficients, supporting the integrity and cohesiveness of the final factor structure.

**Table 8:** Cronbach's alpha coefficients for the total PFAC scale and its subscales.

Dimension	Dimension name	Number of items	Cronbach's alpha	95% CI
1	Ethical and belief‐based responses	10	0.83	0.81–0.85
2	Psychological response	10	0.78	0.76–0.80
3	Psychological reactions of victims	7	0.81	0.79–0.83
Overall	Total	27	0.85	0.84–0.86

*Note:* Cronbach's α with 95% CI (Bonett method); *n* = 100 for reliability subsample. Subscale α: 0.83 (0.81–0.85); 0.78 (0.76–0.80); 0.81 (0.79–0.83). Total α: 0.85 (0.84–0.86).

### 3.6 Finalized PFAC Scale

The final Psychological First Aid Competence (PFAC) Scale consists of 27 positively worded items, each scored on a 5‐point Likert scale (0 = Never to 4 = Always). Total possible scores range from 27 to 135, with higher scores indicating greater competence. Table 7 presents the full item list grouped by its three dimensions.

## 4 Discussion

The present study aimed to develop and validate a competency assessment scale for responders providing Psychological First Aid (PFA) during disasters and crises. The final instrument consisted of 27 items across three dimensions and demonstrated satisfactory psychometric properties according to the 2018 COSMIN standards. Overall, the findings indicate that the PFAC scale is a reliable and valid instrument for assessing PFA competence among emergency responders.

Item generation in this study was informed by an extensive review of existing literature as well as qualitative interviews with Iranian emergency responders. This dual deductive–inductive approach enhanced both the theoretical grounding and cultural relevance of the scale. Similar methodological approaches have been used in previous studies, such as the development of the PFA self‐efficacy scale by Kılıç Bayageldi et al. [30]. However, an important distinction is that the Kılıç Bayageldi instrument assesses self‐efficacy—one's belief in the ability to perform PFA—whereas the PFAC developed in this study measures actual competence, including knowledge, skills, and behavioral capability. This conceptual distinction is critical, as competence reflects performance capacity, whereas self‐efficacy reflects perceived ability.

Other instruments, such as the PFA Knowledge and Competency Questionnaire designed by Zhang et al. [31], relied solely on the WHO PFA facilitator manual. According to COSMIN recommendations, item generation that excludes perspectives of the target population risks reducing content relevance and specificity [36, 53]. To overcome this limitation, the present study incorporated a qualitative phase to ensure the inclusion of contextually grounded and practice‐based competencies. This mixed‐methods strategy strengthens the interpretability, cultural fit, and practical utility of the PFAC scale.

A further strength of the scale is its concise structure. The final 27‐item instrument balances comprehensiveness with feasibility, which is essential given the time constraints and high workload typical of emergency responders. Shorter questionnaires have been shown to improve response accuracy and reduce respondent burden [54]. Comparable tools also vary in length: the PFA Knowledge and Competency Questionnaire contains 24 items [31], whereas the PFA Self‐Efficacy Application Scale includes 35 items [30]. The PFAC thus provides a robust yet efficient option for field and training settings.

In this study, the validation process was conducted according to COSMIN standards across five domains: face validity, content validity, construct validity (through EFA), reliability, and internal consistency. These stages were carried out by administering the initial version of the questionnaire designed for emergency responders. All psychometric evaluations yielded satisfactory and acceptable results. For construct validity, 236 participants were selected, with the KMO measure calculated at 0.779. Literature suggests that a sample size of 100 is considered weak, 200 is average, and 300 is optimal; similarly, a KMO value close to 1 is deemed highly sufficient, whereas values below 0.50 are considered unacceptable [30].

For a similar scale, the *PFA Knowledge and Competency Questionnaire*, the researcher did not report the stages of validity assessment, with only internal consistency reported, ranging from 0.79 to 0.83 [31]. In the *PFA Application of the Self‐Efficacy* scale, construct validity (using exploratory and confirmatory factor analyzes), internal consistency, and test‐retest reliability were validated. A noted limitation of this scale was that, despite being intended for aid providers in disaster scenarios, the validation process was conducted on a sample of 600 students [30]. In the present scale, while the sample size for factor analysis was constrained, efforts were made to conduct the validation steps within the same population initially used in the scale's design.

In the present study, three distinct dimensions emerged from exploratory factor analysis: ethical and belief‐based responses, psychological responses, and recognition of psychological reactions in victims, collectively explaining 36.94% of the variance. A review of literature showed that no other scale encompasses these specific three dimensions. The only comparable instrument, the PFA Application of Self‐Efficacy, was validated with a single factor [30].

Of the three factors derived from the present scale, the ethical and belief‐based responses factor exhibited the highest factor loading, suggesting that, within the studied population, this dimension held greater significance compared to the other factors. Given the Iranian religious context, moral and religious considerations are prioritized in all circumstances, even in crises. The emphasis on ethical concerns as a crucial aspect of responder competence may be due to the understanding that neglecting these aspects can lead to discriminatory service provision [55], a reduction in safety, potential violence, and a lack of respect for the culture and religious beliefs of those affected [56]. Additionally, several studies have demonstrated a strong link between attention to culture, religion, beliefs, and psychological dimensions [57, 58] as disrespect toward personal beliefs can provoke violence and conflict [59].

## 5 Conclusion

This study addressed a significant gap in the field by developing a validated and reliable instrument to assess responders' competence in providing Psychological First Aid. The resulting PFAC scale consists of 27 items across three domains—ethical and belief‐based responses, psychological responses, and understanding psychological reactions among victims—and demonstrated satisfactory psychometric properties. The scale offers a structured framework for identifying strengths and areas requiring improvement among responders and provides a valuable resource for planners, trainers, and policymakers to enhance PFA readiness.

One of the key strengths of this study lies in the dedicated design and psychometric validation of this scale, specifically for the responder population. Item generation involved a review of existing literature and scales and qualitative interviews with the responders. The psychometric validation process utilized the latest version of the COSMIN checklist, widely recognized as a credible framework for scale development. It also has a questionnaire that can be generalized to other fields and countries.

However, the study encountered some limitations. In the qualitative phase, there was a possibility that participants might not fully disclose their true experiences. To address this, we conducted interviews with strict confidentiality protocols, ensuring that all data and recordings were securely handled. In cases where participants opted out of recording, their responses were documented manually. Coordinating interviews with responders also posed challenges; thus, interviews were scheduled according to participants' preferred times and locations. For the quantitative phase, online questionnaires were employed due to limitations on in‐person data collection caused by logistical and time constraints. This study represents the initial development and preliminary validation of the PFAC scale, focusing on item generation, face and content validity, exploratory factor analysis, and internal consistency reliability. Due to the high workload and limited availability of emergency responders, it was challenging to recruit a sufficiently large and diverse sample to support additional psychometric testing, such as confirmatory factor analysis or convergent/divergent validity assessments. These analyzes are, therefore, planned for subsequent validation phases with larger and more varied samples.

Due to time limits, additional validation stages—such as convergent validity, test‐retest reliability, and confirmatory factor analysis—were not conducted. It is recommended that future research should explore these aspects to further strengthen the validation of the instrument.

## Author Contributions

**Conceptualization:** Maryam AziziM, Maryam Azizi, and Nahid Rajai. **Methodology:** Maryam AziziM and Maryam Azizi. **Data curation:** Maryam AziziM and Maryam Azizi. **Writing – original draft preparation:** all authors. **Writing – review and Editing:** Maryam AziziM and Maryam Azizi. All authors have read and agreed to the published version of the article.

## Ethics Statement

This study was approved by the Biomedical Research Ethics Committee of Shiraz University of Medical Sciences, Shiraz, Iran (code IR.SUMS.REC.1402.368). The study adhered to the Declaration of Helsinki. Participation was voluntary; written informed consent was obtained from all participants. Anonymity and confidentiality were ensured, participants could contact the research team, and they could withdraw at any time without providing a reason.

## Conflicts of Interest

The authors declare no conflicts of interest.

## Transparency Statement

The lead author Maryam Azizi affirms that this manuscript is an honest, accurate, and transparent account of the study being reported; that no important aspects of the study have been omitted; and that any discrepancies from the study as planned (and, if relevant, registered) have been explained.

## Data Availability

De‐identified datasets generated and/or analyzed during the current study are available from the corresponding author on reasonable request (subject to institutional/ethical approvals to protect participant privacy).
